# Active surveillance inclusion criteria under scrutiny in magnetic resonance imaging-guided prostate biopsy: a multicenter cohort study

**DOI:** 10.1038/s41391-021-00478-2

**Published:** 2021-12-17

**Authors:** Kira Kornienko, Fabian Siegel, Angelika Borkowetz, Manuela A. Hoffmann, Martin Drerup, Verena Lieb, Johannes Bruendl, Thomas Höfner, Hannes Cash, Jost von Hardenberg, Niklas Westhoff, GESRU Academics Prostate Cancer Group, Jost von Hardenberg, Burkhard Beyer, Johannes Bründl, Johannes Cash, Jonas Herrmann, Jan Philipp Radtke, Manuela A. Hoffmann, Conrad Leitsmann, Kira Kornienko, Thomas Worst, Hendrik Borgmann, Johannes Linxweiler, Niklas Klümper, Mike Wenzel, Maria Noemi Welte, Niklas Westhoff, Christoph Würnschimmel, August Sigle, Samy Mahjoub, Gernot Ortner, Jeremy Kwe, Sven-Thorben Langenberger

**Affiliations:** 1 Department of Urology, Charité University Medicine Berlin, Berlin, Germany; 2 Division of Epigenomics and Cancer Risk Factors, German Cancer Research Center (DKFZ), Heidelberg, Germany; 3 Department of Biomedical Informatics at the Center for Preventive Medicine and Digital Health, Medical Faculty of Mannheim, University of Heidelberg, Mannheim, Germany; 4 Department of Urology and Urosurgery, University Medical Center Mannheim, Medical Faculty Mannheim, University of Heidelberg, Mannheim, Germany; 5 Department of Urology, University Hospital Carl Gustav Carus, Technische Universität Dresden, Dresden, Germany; 6 Department of Occupational Health and Safety, Federal Ministry of Defense, Bonn, Germany; 7 Department of Nuclear Medicine, University Medical Center of the Johannes Gutenberg University Mainz, Mainz, Germany; 8 Department of Urology, Paracelsus Medical University, Salzburg, Austria; 9 Department of Urology and Pediatric Urology, University Hospital Erlangen, Friedrich-Alexander-University Erlangen-Nürnberg, Erlangen, Germany; 10 Department of Urology, Caritas St. Josef Medical Center, University of Regensburg, Regensburg, Germany; 11 Department of Urology, University Medical Center of the Johannes Gutenberg University Mainz, Mainz, Germany; 12 PROURO Berlin, Berlin, Germany; 13 Department of Urology, University Magdeburg, Magdeburg, Germany; 14 Department of Urology and Urosurgery, Medical Faculty Mannheim, Heidelberg University, Mannheim, Germany; 15 Department of Urology, Martini-Klinik, University-Clinic Hamburg Eppendorf, Hamburg, Germany; 16 Department of Urology, University Hospital Essen, Essen, Germany; 17 Department of Urology, University Medical Center Göttingen, Göttingen, Germany; 18 Department of Urology and Pediatric Urology, Saarland University, Homburg/Saar, Germany; 19 Department of Urology and Pediatric Urology, University Medical Center Bonn, Bonn, Germany; 20 Department of Urology, University Hospital Frankfurt, Frankfurt am Main, Germany; 21 Department of Urology, Faculty of Medicine, Medical Centre - University of Freiburg, Freiburg, Germany; 22 Department of Urology, Uro-Oncology, Robot-assisted and Specialized Urologic Surgery, Cologne University Hospital, Cologne, Germany; 23 Department of Urology and Andrology, General Hospital Hall in Tirol, Hall in Tirol, Austria

**Keywords:** Prostate cancer, Prostate cancer

## Abstract

**Background:**

Although multiparametric magnetic resonance imaging (mpMRI) is recommended for primary risk stratification and follow-up in Active Surveillance (AS), it is not part of common AS inclusion criteria. The objective was to compare AS eligibility by systematic biopsy (SB) and combined MRI-targeted (MRI-TB) and SB within real-world data using current AS guidelines.

**Methods:**

A retrospective multicenter study was conducted by a German prostate cancer (PCa) working group representing six tertiary referral centers and one outpatient practice. Men with PCa and at least one MRI-visible lesion according to Prostate Imaging Reporting and Data System (PI-RADS) v2 were included. Twenty different AS inclusion criteria of international guidelines were applied to calculate AS eligibility using either a SB or a combined MRI-TB and SB. Reasons for AS exclusion were assessed.

**Results:**

Of 1941 patients with PCa, per guideline, 583–1112 patients with PCa in both MRI-TB and SB were available for analysis. Using SB, a median of 22.1% (range 6.4–72.4%) were eligible for AS. Using the combined approach, a median of 15% (range 1.7–68.3%) were eligible for AS. Addition of MRI-TB led to a 32.1% reduction of suitable patients. Besides Gleason Score upgrading, the maximum number of positive cores were the most frequent exclusion criterion. Variability in MRI and biopsy protocols potentially limit the results.

**Conclusions:**

Only a moderate number of patients with PCa can be monitored by AS to defer active treatment using current guidelines for inclusion in a real-world setting. By an additional MRI-TB, this number is markedly reduced. These results underline the need for a contemporary adjustment of AS inclusion criteria.

## Introduction

Prostate cancer (PCa) diagnostics have changed significantly in recent years so that clinically significant PCa (csPCa) are detected earlier, but also the risk of overdiagnosis of insignificant PCa increases with it [1]. To avoid overtreatment and to defer active treatment, Active Surveillance (AS) is incorporated as a standard option in patients with localized PCa in guidelines worldwide [2]. Since the strategy of AS was first described in 2002, heterogeneity in definitions and patient selection remains controversial in the literature, centers, and guidelines among different countries [3, 4]. Current AS inclusion criteria and follow-up are traditionally based on prostate-specific antigen (PSA), digital rectal examination (DRE), number of cancer-infiltrated biopsy cores, tumor infiltration per biopsy core, and the Grading Group (GG). Recent guidelines recommend prostate imaging by multiparametric magnetic resonance imaging (mpMRI) in primary cancer diagnosis followed by targeted biopsies (TB) in addition to systematic biopsies (SB) to identify men with csPCa accurately [2, 5, 6]. Also, mpMRI adds value to the entry criteria and follow-up guidance in men under AS [7].

However, although some international guidelines already recommend MR-imaging for AS selection, the criteria for AS inclusion are based on SB [5, 8]. Due to increased detection of csPCA and multiple TB per lesion, combined MRI-TB and SB will likely exclude patients from AS eligibility if the selection is based on traditional criteria [9].

For this reason, a study demonstrating how many patients disqualify for current AS criteria by the inclusion of MR-imaging in cancer diagnostics is urgently needed. This multicenter cohort analysis compares the number of patients eligible for AS according to relevant international AS guideline recommendations between SB and a combined MRI-TB and SB approach. We aim to pave the way to assess new definitions for AS eligibility within future trials by taking MRI parameters into account.

## Subjects and methods

The study cohort was conducted within a multicenter project of a German prostate cancer working group (German Society of Residents in Urology Academics). It is composed of 1941 patients from six tertiary referral centers and one outpatient urologist`s office. All German centers belong to the German Cancer Society (Deutsche Krebsgesellschaft, DKG)-certified PCa centers as previously described [10]. All registered patients had a confirmed PCa by combined MRI-TB and SB. Patients without detection of PCa were not recorded for this analysis. This analysis was approved by the local ethics committees (lead investigator center Mannheim: 2018-878R-MA).

### MR imaging and biopsy

A mpMRI was performed in all patients before biopsy. Board-certified radiologists read and interpreted the MRI according to PI-RADSv2 in all centers without a central review [11]. Patients with at least one MRI-visible lesion were included for analysis. Board-certified urologists or residents under supervision performed a software-based transrectal (six centers) or transperineal (one center) MRI-TB and SB. According to consensus recommendations, pathological processing was done and enabled a separate appraisal of each TB and SB core.

### Active surveillance inclusion criteria

A comprehensive non-systematic review (MEDLINE via PubMed and websites of international guidelines) was performed to identify international PCa guidelines recommending AS inclusion criteria. After collection, we selected 13 current guidelines with 22 different AS criteria for application, published between 2013 and 2020 (Table 1) [2, 5, 6, 8, 12–20]. These guidelines were accessed in June 2021. All guidelines include information regarding the maximum GG, the maximum PSA, and the maximum clinical stage as eligibility criteria for AS. Some guidelines also include a maximum number of cancer-positive cores, a maximum cancer core infiltration, and PSA density (PSAD). Due to the different information available from the participating centers, a digital rectal examination (DRE) was considered either normal or suspicious. The recently updated PCFA and EAU inclusion criteria for intermediate risk PCa include a maximum Gleason 4 pattern of <10%. Since this information was not given in our cohort, we did not include these criteria for further analyses.

**Table 1:** Current Active Surveillance protocols of selected guidelines (all based on systematic biopsies, none includes mpMRI).

	Guideline	Risk category	Max. GG	Max. PSA serum (ng/ml)	Max. positive cores (n)	Max. extent cancer per core (%, mm)	Max. clinical stage	PSAD (ng/ml/ cm^3^)
Asia	NCCS	Low	1	<10	2	≤50%	cT2a	<0.15
Australia	PCFA	Low	1	≤20			cT2c	
		Intermediate^a^	2, GS 4 pattern <10%	<10			cT2a	
Belgium	KCE	Low	1	<10			cT2a	
Canada (Ontario)	CCO	Low	1	<10	2	≤50%	cT2a	
Europe	EAU	Low	1	≤10			cT2a	
		Intermediate^a^	2, GS 4 pattern <10%	≤10			cT2a	
	ESMO	Low	1	<10			cT2a	
		Intermediate	3	10–20			cT2b	
Finland	FCCG	Low	1	<10	2		cT2b	
Great Britain	NICE	Low	1	<10			cT2a	
		Intermediate	3	10–20			cT2b	
Germany	GSU	Low	1	≤10	2	≤50%	cT2a	
Spain	I+CS	Low	1	≤10		<50%	cT2a	
		Intermediate	2	≤15		<50%	cT2a	
The Netherlands	DUA	Low	1	<10	2		cT2a	
United States of America	NCCN	Very low	1	<10	2	≤50%	cT1c	<0.15
		Low	1	<10			cT2a	
		Favorable intermediate	2	10-<20		≤50%	cT2c	
	AUA	Very low	1	≤10	<34%	≤50%	cT2a	<0.15
		Low	1	<10			cT2a	
		Favorable intermediate	2	10-<20			cT2c	

*GG* gleason grade, *PSA* prostate specific antigen, *PSAD* prostate specific antigen density.

^a^not considered for subsequent analyses.

*AUA* American Urological Association [2], *CCO* Cancer Care Ontario [12], *DUA* Dutch Urological Association [13], *EAU* European Association of Urology [6], *ESMO* European Society for Medical Oncology [14], *FCCG* The Finnish Medical Society Duodecim [15], *GSU* German Society of Urology [5], *I+CS* Aragon Institute of Health Sciences [16], *KCE* Belgian Healthcare Knowledge Centre [17], *NICE* National Institute for Health and Clinical Excellence [8], *NCCN* The National Comprehensive Cancer Network [18], *NCCS* National Cancer Centre Singapore [19], *PCFA* Prostate Cancer Foundation of Australia [20].

### Statistical analyses

The primary outcome was to compare AS eligibility between SB and combined MRI-TB and SB according to contemporary international guideline AS selection criteria. Each single AS definition was applied to all patients for whom every required clinical (PSA, DRE) or histopathological information (GG, number of biopsy cores, cancer core infiltration in % or millimeter) was available from the dataset. Patients with incomplete data were excluded from the single analysis per AS definition. Patients with PCa in both SB and MRI-TB were selected for subsequent analyses. For every AS definition, we then calculated the number of patients meeting the particular inclusion criteria if virtually only SB would have been obtained as well as the number of patients if both MRI-TB and SB would have been obtained. The reasons for exclusion from AS due to additional MRI-TB were assessed for every patient per definition. Every single SB and MRI-TB core was counted for the total number of (cancer-positive) cores per biopsy.

Continuous variables were described using medians and interquartile ranges (IQR), whereas categorical variables were characterized using proportions. Confidence intervals were estimated based on 10,000 stratified bootstrap samples with replacement for sample sizes of 100. Sampling was stratified based on Gleason Score. Eligibility rates were compared using a binominal test. A *p* < 0.05 was considered significant. Python 3.8.10 with libraries scikit-learn 1.0.1, SciPy 1.7.1 and pandas 1.3.4 was used for bootstrapping and binominal testing.

## Results

### Patient characteristics

**Fig. 1:**
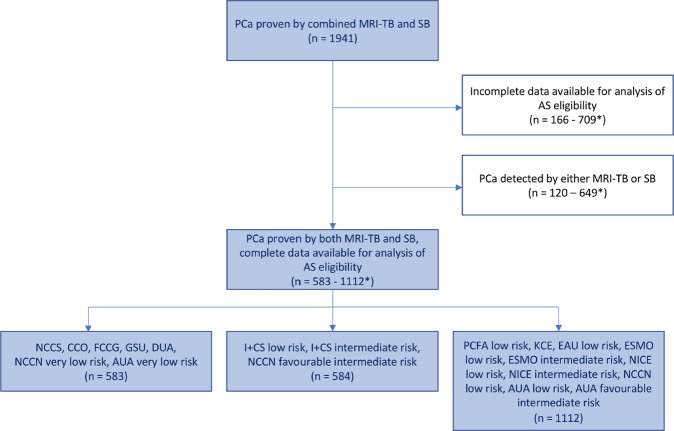
Summary of patient selection. *Number of analyzable patients dependent on available data per guideline definition. AS active surveillance, PCa prostate cancer, MRI-TB magnetic resonance imaging - targeted biopsy, SB systematic biopsy.

The total cohort consisted of 1941 patients who had PCa proven by a combined MRI-TB and SB. Demographic data, results of mpMRI and consecutive biopsies are presented in Table 2. For subsequent analyses on AS eligibility, per guideline definition, between 583 and 1112 patients with PCa in MRI-TB and SB were available, depending on the patient data required to meet the criteria (Fig. 1).

**Table 2:** Demographic, imaging and biopsy data of 1941 patients who received a combined MRI-targeted and systematic biopsy.

	*n* or median	IQR or %
Patient characteristics		
Age (years)	68.8	(63–73.7)
PSA (ng/ml)	8.1	(6–11.6)
DRE suspicious	336	17.3
Prostate volume (ml)	44.8	(33–60)
PSAD (ng/ml/cm^3^)	0.18	(0.12–0.24)
Biopsy data		
TB cores total	4	(2–5)
TB cores cancer	2	(1–3)
SB cores total	12	(10–12)
SB cores cancer	2	(1–4)
PCa in TB and SB	1207	62.2
PCa in TB only	306	15.8
PCa in SB only	382	19.7
Imaging data		
Index lesion suspicious	125	6.4
PI-RADS Index <3	40	2.1
PI-RADS Index 3	259	13.3
PI-RADS Index 4	886	45.7
PI-RADS Index 5	631	32.5

*DRE* digital rectal examination, *PCA* prostate cancer, *PI-RADS* prostate imaging reporting and data system, *PSA* prostate specific antigen, *PSAD* PSA-density, *SB* systematic biopsy, *TB* targeted biopsy.

### AS eligibility using systematic biopsy

**Fig. 2:**
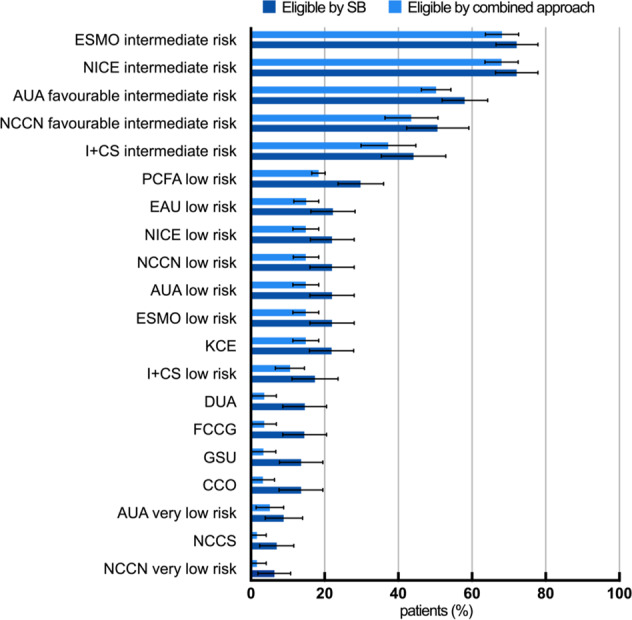
Active surveillance eligibility. Shown are the median eligibility rates (with 95% CI) per guideline inclusion criteria. Light blue bars represent eligibility by a single systematic biopsy, dark blue bars represent eligibility by a combined MRI-TB and SB.

AS could be applied to a median of 22.1% of patients with PCa if virtually only a SB would have been obtained. The range was 6.4–72.4%, depending on guideline definitions. Among low risk AS criteria, a median of 17.5% (6.4–29.9%) patients were eligible. Among intermediate risk AS criteria, a median of 58% (44.4–72.4%) were eligible. The lowest inclusion rates were detected for the NCCN very low risk criteria, whereas the highest inclusion rates were achievable by the NICE and ESMO intermediate risk criteria (Fig. 2).

### AS eligibility using MRI-targeted and systematic biopsy

Using combined MRI-TB and SB, a median of 15% (1.7–68.3%) patients had PCa eligible by one of the AS inclusion criteria. Median eligibility was 10.6% (1.7–18.4%) among low risk AS criteria and 50.1% (37.5–68.3%) among intermediate risk criteria. The lowest inclusion rates were detected for the NCCS and NCCN very low risk criteria and the highest inclusion rates for the NICE and ESMO intermediate risk criteria. The addition of an MRI-TB led to an overall loss of eligibility for a median of 7.1% patients (range 3.8–11.4%), corresponding to a 32.1% reduction compared to the eligibility by SB (Fig. 2). This reduction was statistically significant in the AS criteria of PCFA low risk (*p* = 0.018), FCCG (*p* = 0.005), DUA (*p* = 0.005), GSU (*p* = 0.007) and CCO (*p* = 0.005) (Table 3).

**Table 3:** Comparison of guideline dependent Active Surveillance eligibility of patients with PCa proven by combined MRI-targeted biopsy and systematic biopsy.

Guideline	Type of biopsy	Number of patients	AS eligible patients (mean)	95% CI	*p* value
ESMO intermediate risk	systematic	1112	72.2	66.5–78.0	
	combined	1112	68.1	63.6–72.7	0.227
NICE intermediate risk	systematic	1112	72.2	66.5–77.9	
	combined	1112	68.1	63.6–72.6	0.226
AUA favourable intermediate risk	systematic	1112	58.1	52.0–64.3	
	combined	1112	50.3	46.3–54.3	0.093
NCCN favourable intermediate risk	systematic	584	50.7	42.3–59.2	
	combined	584	43.6	36.5–50.8	0.115
I+CS intermediate risk	systematic	584	44.2	35.4–52.9	
	combined	584	37.4	29.9–44.9	0.124
PCFA low risk	systematic	1112	29.9	23.7–36.0	
	combined	1112	18.4	16.6–20.1	0.018
EAU low risk	systematic	1112	22.3	16.3–28.3	
	combined	1112	15.1	11.7–18.5	0.078
NICE low risk	systematic	1112	22.1	16.1–28.1	
	combined	1112	15.0	11.5–18.5	0.079
NCCN low risk	systematic	1112	22.1	16.1–28.1	
	combined	1112	15.0	11.5–18.4	0.080
AUA low risk	systematic	1112	22.1	16.1–28.1	
	combined	1112	15.0	11.5–18.5	0.080
ESMO low risk	systematic	1112	22.1	16.0–28.1	
	combined	1112	15.0	11.6–18.4	0.080
KCE	systematic	1112	22.0	16.0–28.0	
	combined	1112	14.9	11.4–18.5	0.082
I+CS low risk	systematic	584	17.4	11.1–23.7	
	combined	584	10.6	6.7–14.6	0.073
DUA	systematic	583	14.7	8.7–20.6	
	combined	583	3.6	0.3–7.0	0.005
FCCG	systematic	583	14.6	8.7–20.6	
	combined	583	3.6	0.3–7.0	0.005
GSU	systematic	583	13.6	7.8–19.5	
	combined	583	3.5	0.2–6.8	0.007
CCO	systematic	583	13.6	7.7–19.5	
	combined	583	3.3	0.1–6.5	0.005
AUA very low risk	systematic	583	9.0	4.0–14.0	
	combined	583	5.2	1.5–8.9	0.167
NCCS	systematic	583	7.0	2.4–11.7	
	combined	583	1.7	0–4.2	0.053
NCCN very low risk	systematic	583	6.4	2.0–10.8	
	combined	583	1.7	0–4.2	0.073

*AUA* American Urological Association [2], *CCO* Cancer Care Ontario [12], *DUA* Dutch Urological Association [13], *EAU* European Association of Urology [6], *ESMO* European Society for Medical Oncology [14], *FCCG* The Finnish Medical Society Duodecim [15], *GSU* German Society of Urology [5], *I+CS* Aragon Institute of Health Sciences [16], *KCE* Belgian Healthcare Knowledge Centre [17], *NICE* National Institute for Health and Clinical Excellence [8], *NCCN* The National Comprehensive Cancer Network [18], *NCCS* National Cancer Centre Singapore [19], *PCFA* Prostate Cancer Foundation of Australia [20].

### Reasons for ineligibility

Table 4 depicts why patients were ineligible for AS per protocol when MRI-TB was added to SB. Thereby, an MRI-TB can affect the GG, the number of cores, and the cancer core infiltration, whereas PSA and PSAD remain impaired. Regarding these three criteria, in nine AS guideline definitions, the GG was the only inclusion criterion. Thus, a higher GG detected by MRI-TB compared to the GG detected by SB, which exceeded the inclusion criterion, was the only reason for exclusion in all of these AS definitions.

**Table 4:** Distribution of exclusion criteria (GG, number of cores, cancer core infiltration) among patients who were Active Surveillance ineligible when MRI-targeted biopsy was added to systematic biopsy.

Guideline	Excluded patients by any AS criterion (%)	Criterion for exclusion (% of all excluded patients per guideline)						
		Max. GG only	Max. cores only	Max. infiltration only	Max. GG & cores	Max. GG & infiltration	Max. cores & infiltration	Max. GG & cores & infiltration
NCCS	5.3	3.2	48.4	0	22.6	0	12.9	12.9
CCO	10.3	8.3	38.3	1.7	21.7	0	10.0	20.0
FCCG & DUA	11.0	7.8	51.6	-	40.6	-	-	-
GSU	10.1	8.5	39.0	0.0	22.0	0.0	10.2	20.3
I+CS low risk	6.8	57.5	-	0	-	42.5	-	-
I+CS high risk	6.8	52.5	-	0	-	47.5	-	-
NCCN very low risk	4.6	3.7	48.1	0	22.2	0	14.8	11.1
NCCN favourable intermediate risk	7.2	57.1	-	0	-	42.9	-	-
AUA very low risk	3.8	36.4	0	18.2	4.5	22.7	9.1	9.1
PCFA low risk, KCE, EAU low risk, ESMO low risk, NICE low risk, NICE intermediate risk, NCCN low risk, AUA low risk, AUA favourable intermediate risk	4.1–11.4	100	-	-	-	-	-	-

*AS* active surveillance, *GG* gleason grade.

*AUA* American Urological Association [2], *CCO* Cancer Care Ontario [12], *DUA* Dutch Urological Association [13], *EAU* European Association of Urology [6], *ESMO* European Society for Medical Oncology [14], *FCCG* The Finnish Medical Society Duodecim [15], *GSU* German Society of Urology [5], *I+CS* Aragon Institute of Health Sciences [16], *KCE* Belgian Healthcare Knowledge Centre [17], *NICE* National Institute for Health and Clinical Excellence [8], *NCCN* The National Comprehensive Cancer Network [18], *NCCS* National Cancer Centre Singapore [19], *PCFA* Prostate Cancer Foundation of Australia [20].

If the maximum number of cancer-positive biopsy cores represented one of the AS inclusion criteria, exceeding this cut-off by additional MRI-TB was the most frequent exclusion criterion (a median of 48.2% (2.1–55.4%) of all excluded patients per guideline).

Moreover, a median of 12.9% of patients (0–36.6%) did not match AS criteria due to a combination of two or three exclusion criteria (GG, number of cores, cancer core infiltration).

## Discussion

AS is a widely applied management option for patients with localized PCa. Still, inclusion criteria for AS vary between guidelines worldwide due to a lack of data from prospective randomized controlled trials. Although mpMRI is now considered standard in primary diagnostics and is already recommended for AS selection and monitoring in some guidelines, it is not incorporated in guideline-based criteria that define a patient to be eligible for AS.

This study demonstrates how many patients qualify for current AS criteria in a multicenter cohort of real-world data: when comparing 20 international guideline definitions, we found that (i) only a moderate number of patients with biopsy-proven PCa qualified for AS by SB and (ii) by inclusion of MRI-TB, almost one third further disqualified for AS. Patients dropped out due to either an upgrading in GG, a higher number of cancer-positive cores, a higher percentage of infiltration, or a combination of these factors. (iii) The variability in AS inclusion criteria generates a tremendous range of eligible patients.

The major task of current PCa diagnostics is to accurately and early detect csPCa. With the introduction of prostate mpMRI to visualize cancer lesions and perform TB, PCa diagnostics have markedly changed [11]. Prospective randomized trials demonstrated an improved csPCa detection, but up to 10% might be missed [21]. Hence, a TB of suspicious MRI-lesions still requires a combined approach including SB. This is at the expense of concurrent detection of insignificant PCa with an indolent clinical course [22]. Radical prostatectomy or radiation therapy is commonly considered as overtreatment for insignificant PCa [23]. AS is therefore increasingly proposed to defer or avoid active treatment in insignificant (low risk) disease. Our study reveals that many patients with insignificant PCa are not eligible for AS based on current guideline recommendations. Also, comparative analysis shows that eligibility largely depends on the underlying AS inclusion criteria. Whereas only 3% of patients matched the criteria of the GSU guideline, in 68% treatment could be deferred when applying the ESMO and NICE intermediate-risk guidelines. These results demonstrate that standardization of AS guidelines is required even when only a SB was obtained at baseline.

The addition of an MRI-TB at baseline or confirmatory biopsy further reduces AS eligibility but also improves safety for patients who defer active treatment. The complementary effect of MRI-TB and SB in AS is proven in the primary as well as in the follow-up biopsy setting. The ASIST trial showed that mpMRI at baseline before the confirmatory biopsy results in significantly fewer AS failure rates [7]. Moreover, MRI-visible disease at baseline is associated with a shorter time to active cancer treatment [24]. In a meta-analysis, more patients with a positive MRI were upgraded at confirmatory biopsy (35% vs. 12%). This analysis also revealed that the tumor was upgraded by nearly the same percentage by additional MRI-TB (7%) and SB (10%), supporting the combined approach for maximized cancer detection [25].

However, we found that the combined biopsy also leads to a 32.1% reduction of patients matching the AS inclusion criteria compared to SB. Again, reflecting the wide variability, the largest absolute reduction was seen in the PCFA low risk definition (−11.5%), whereas 3.8% less patients were AS candidates using the AUA very low risk definitions.

Two major effects of an additional MRI-TB were identified that decrease AS eligibility. First, GG upgrading was the most frequent reason for disqualification. Consequently, on the one hand, csPCa were more precisely identified compared to the single SB. On the other hand, AS for intermediate risk PCa is still under debate. In the original Epstein criteria, AS was offered to patients with a GG 1 exclusively [26]. In a study of 259 men who underwent an MRI-TB and SB for follow-up in AS, many men whose pathology exceeded the original Epstein criteria remained stable for up to four years of surveillance. However, the incidence ratio of upgrading during AS of men with GG 2 compared to GG 1 was 4.25 [27]. In the SPCG-4 study, there was no associated death after 29 years in patients who received radical prostatectomy with a secondary Gleason pattern 4 of ≤10% in the prostatectomy specimen [28]. Due to the diagnosis of higher GG by the additional MRI-TB as shown by our analysis, an expansion of existing conservative AS protocols which include patients with Gleason pattern 4 should be considered. Otherwise, the role of AS in the therapy algorithm of PCa will further decrease. Intermediate-risk patients should, nonetheless, receive a strict follow-up.

Second, another important finding of our study is that criteria of the maximum cancer extent eligible for AS have to be redefined in the era of additional MRI-TB. Cancer volume in the prostate is an important indicator for progression and is defined by a tumor volume of 0.5 cm3 [26, 29]. The traditional maximum number of positive cores cannot be adopted when targeting a single lesion several times, especially, since multiple targeting per lesion is increasingly recommended [30]. On the contrary, the maximum cancer core length obtained by TB of a suspicious MRI lesion was shown to directly correlate to the index cancer volume and might therefore better predict csPCa and AS eligibility [31].

As a consequence of the lack of high-level evidence, the DETECTIVE collaborative study developed consensus criteria based on the criteria most often published which include: GG 1, clinical stage cT2a, PSA < 10 ng/ml, and PSAD <0.15 ng/ml/cm^3^ [32]. When applying these criteria to our cohort, only 11.1% of all patients were eligible for AS by a single SB and 7.7% by a combined biopsy. DETECTIVE also recommends considering GG 2 cancer with a low number of positive cores for deferred treatment [32]. There was no consensus on the maximum tumor extent based on the number of cancer-positive cores, cancer infiltration or cancer volume on mpMRI. However, the study proposes that if TBs are performed, instead of the number of positive cores, the number of positive sextants and the MRI index lesion volume should be considered indicators of cancer extent [32]. In contrast to DETECTIVE, Nassiri et al. showed that cancer core length, core infiltration, and the number of positive cores were not associated with a higher risk of reclassification [27].

Thus, thresholds remain contentious to which disease extent on biopsy ought to lead to exclusion [33]. Therefore, mpMRI and TB should be integrated into AS inclusion criteria. When defining new cut-off values, the outcome of multiple cancer positive TB`s per lesion and the shift of grading must be considered with caution. A more liberal entry approach allows more patients to defer the negative impact of active treatments but requires more frequent and strict surveillance [34]. Addition of new biomarkers shows potential to further improve characterization of more aggressive PCa. For example, mutations of DNA damage repair genes like BRCA2 are associated with an 8.6 times higher risk to develop an aggressive early-onset PCa (<65 years) [35].

Besides the strengths of this study, some limitations have to be addressed. First, due to our multicenter cohort and retrospective data documentation, suspicious clinical stages cannot be further distinguished, which has been seen as notable differences in guidelines. Second, data is collected from different centers, with various levels of radiological and urological experience and different biopsy standards. A central MRI review was not available. Nevertheless, these real-world data are of the highest importance to decide on new AS inclusion criteria. Third, a lack of long-term follow-up as well as pathology reports from prostatectomy specimen does not allow us to develop an adjusted version of AS criteria and estimate the effect of these results on patient outcome.

In conclusion, incorporation of additional MRI-TB in primary PCa revolutionized diagnostics and risk stratification. However, although it is now recommended for primary and re-biopsy, it is not yet part of AS inclusion criteria. This analysis underlines that combined MRI-TB and SB markedly reduces patients` eligibility for AS using current international AS protocols that are still based on SB results. This is mostly due to a higher GG and number of cancer-infiltrated cores. In the light of current uncertainty on the relevance of this grade-shift for patient outcomes, we advocate to re-define AS inclusion criteria and to initiate future studies assessing the effect of upgrading by MRI-TB and validating adjusted AS criteria that contain imaging parameters.

In addition, new biomarkers, like serum and tissue markers, might be included in future protocols to improve identification of AS candidates.
